# The Impact of Environmental and Housing Factors on the Distribution of Triatominae (Hemiptera, Reduviidae) in an Endemic Area of Chagas Disease in Puebla, Mexico

**DOI:** 10.3390/diseases12100238

**Published:** 2024-10-02

**Authors:** Miguel Ortega-Caballero, Maria Cristina Gonzalez-Vazquez, Miguel Angel Hernández-Espinosa, Alejandro Carabarin-Lima, Alia Mendez-Albores

**Affiliations:** 1 Centro de Química-ICUAP-Posgrado en Ciencias Ambientales, Benemérita Universidad Autónoma de Puebla, Puebla 72570, Mexico; miguel.ortegacaballero@viep.com.mx (M.O.-C.); miguel.hernandez@correo.buap.mx (M.A.H.-E.); 2 Facultad de Ciencias Biológicas, Benemérita Universidad Autónoma de Puebla, Puebla 72570, Mexico; cristina.gonzalezvazquez@correo.buap.mx; 3 Centro de Investigaciones en Ciencias Microbiológicas, Instituto de Ciencias, Benemérita Universidad Autónoma de Puebla, Puebla 72570, Mexico

**Keywords:** *Trypanosoma cruzi*, Chagas disease, vectors, triatomines, distribution factors, vector-borne disease, public health, neglected tropical diseases, *Triatoma* spp.

## Abstract

Background: Chagas disease (CD), a Neglected Tropical Disease caused by *Trypanosoma cruzi*, affects millions of people in Latin America and the southern US and spreads worldwide. CD results from close interactions between humans, animals, and vectors, influenced by sociodemographic factors and housing materials. Methods: This study aimed to evaluate how these factors, along with seasonal changes, affect the distribution of CD vectors in an endemic community near Puebla, Mexico, using a cross-sectional survey. A total of 383 people from this area, known for the presence of major vectors such as *Triatoma barberi* and *Triatoma pallidipennis*, were surveyed. Results: As a result of the survey, it was found that only 27.4% of respondents knew about CD, and 83.3% owned potential reservoir pets; additionally, the quality of the wall, roof, and floor significantly influenced vector sightings, while the seasonal pattern showed less of an association. Chi-square tests confirmed these associations between vector sightings and housing materials (*p* < 0.001); vector sightings versus seasonal patterns showed less of an association (*p* = 0.04), and land use changes did not show an association (*p* = 0.27). Conclusions: Construction materials play an important role in the sighting of triatomines in homes, so important actions should be taken to improve homes. However, further experimental or longitudinal studies are needed to establish causality.

## 1. Introduction

Chagas disease (CD), caused by the protozoan parasite *Trypanosoma cruzi*, is a tropical parasitic disease that affects millions of people in Latin America and the southern part of the United States, where it is endemic. Furthermore, due to globalization, positive cases have also been confirmed in Western European countries, Japan, Australia, and Canada [1,2].

CD is classified as a Neglected Tropical Disease (NTD) by the World Health Organization. These NTDs are a diverse group of diseases caused by a variety of pathogens, including parasites, and are associated with devastating health, social, and economic consequences. These diseases are predominantly prevalent among impoverished communities in tropical areas, although some have a much wider geographic distribution, receiving limited medical attention and resources for prevention and treatment. The current treatments available for NTDs have limitations in terms of their safety and efficacy, and, more importantly, no new drugs or treatments have been developed since 1970 [3]. Furthermore, even though NTDs should be a priority in pharmaceutical companies’ research and development programs, there is a clear lack of interest because NTDs are not an attractive market in which to make a significant investment [4]. Thus, NTDs are considered to violate human rights and cause marginalization in society [5]. Therefore, one of the key challenges in the control of these diseases is understanding the influence of sociodemographic factors that affect their prevalence and spread [6].

CD is a clear example of an anthropozoonosis, involving close interactions among humans, animal reservoirs, and their vectors, the blood-feeding bugs of the genus Triatominae (Reduviidae, order: Hemiptera), which maintain the transmission cycle [7]. When these bugs feed on an infected reservoir, they acquire the parasite that can then be transmitted to a healthy person or animal through the contamination of micro-wounds in the skin with the bug’s feces when it defecates while feeding [8].

Although an underestimation in the current prevalence of people with Chagas is recognized, with less than 10% of carriers being properly diagnosed [6], an increase in positive cases in urban areas can be predicted [9]. This can be attributed to the effects of anthropic activities such as deforestation, ecosystem invasion, growing urbanization, the construction of precarious housing, the migration of rural populations to urban environments, and globalization. These activities can create favorable conditions for the proliferation of the CD vector and, with this, the concomitant exposure of people to this disease, even in areas that are not considered endemic [10]. Climate change is another important factor that can alter the natural distribution of this vector. As an example, it has been demonstrated that the vector of Lyme disease, which is an arthropod (as in the case of CD), undergoes greater dispersion outside of its ecosystem with a change in the average temperature or the concurrence of shorter and warmer winters [11,12,13]. In Mexico, there are currently no reports or studies documenting significant variations in the population dynamics of vector-borne diseases.

On the other hand, recent studies established a positive correlation between a higher risk of contracting CD and economic vulnerability in individuals. This is attributed to the characteristics of housing construction materials and the ability of CD vectors to infest houses. Furthermore, certain species of vectors show adaptive capabilities in urban areas, contributing to the increased risk in urban settings [5,14].

In this study, various parameters influencing the distribution of triatomines in an endemic community of this disease in a community near the capital city of Puebla in Mexico were determined. Several factors such as housing construction materials, urbanization of the study area, and seasonal changes related to the sighting of CD vectors within households and in the surrounding areas of the homes of residents were evaluated.

## 2. Materials and Methods

### 2.1. Study Area

**Figure 1:**
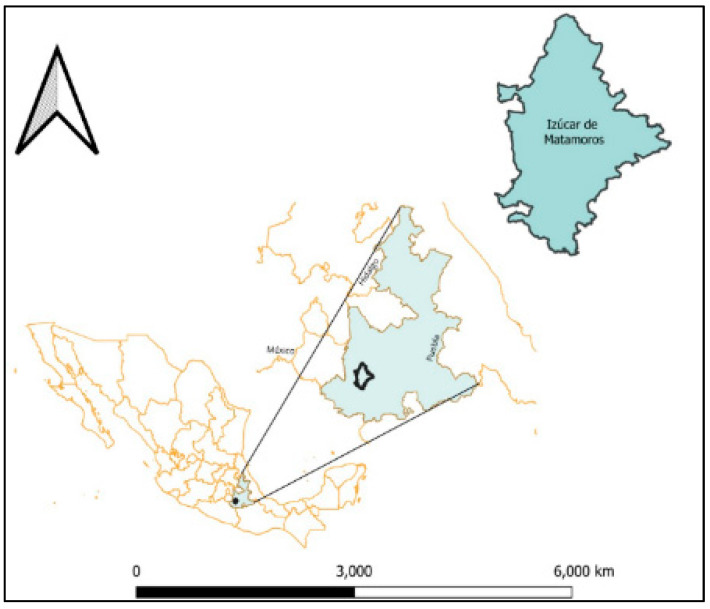
Map showing the location of the Chagas disease endemic area where the surveys were conducted in the state of Puebla, Mexico. The map was designed in QGIS Development Team (2023), QGIS Desktop 3.28.10, Open Source Geospatial Foundation Project: https://qgis.org/es/site/ (accessed on 14 February 2024).

A descriptive and inferential cross-sectional study was conducted in the town of Izucar de Matamoros (18° 36′ 08″ N, 98° 27′ 55″ W), in Puebla, Mexico (Figure 1). Izucar de Matamoros has a population of 82,809 inhabitants, representing a growth of 13.8% compared to the last population census in the year 2010 [15].

The study area is considered endemic because of the presence *Triatoma barberi* and *Triatoma pallidipenis*, along with environmental conditions that favor their survival and distribution [16,17].

### 2.2. Survey Design

In the present study, an easy-to-understand survey was developed. The questions were classified into both polytomous and dichotomous formats based on the required categories to comprehensively address the objective of collecting information. This approach included obtaining informed consent, along with a total of 19 questions, which also encompassed aspects such as age and location in the study area (Supplemental Figure S1). Various aspects were covered, including control-related questions, socioeconomic aspects related to housing, seasons of the year, and the environmental impact associated with land-use change. Specifically, the objective was to establish significant relationships and correlations between these factors and the presence of vector-identified CD.

In the context of the test of independence of variables using the chi-square statistic, socioeconomic questions were formulated to allow for the classification of categories based on observed frequencies. These categories, identified as “A”, “B”, and “C”, are defined in Table 1.

The socioeconomic categories (1, 2, and 3) included intradomiciliary sightings, while those related to changes in land use (4 and 5) considered both intradomiciliary sightings and those in the vicinity of residences. The statistical evaluation relied on Cramer’s coefficient, which quantifies the intensity of associations between the variables under analysis.

### 2.3. Sample and Criterion Selection

A survey was conducted with a total of 383 individuals to achieve a 95% confidence level and a 5% margin of error. This sample was randomly selected from the target population, ensuring data representativeness and the validity of the study’s conclusions.

Participants were required to read and have no visual impairments, as three specific questions in the survey necessitated the ability to identify images of CD vectors. The survey was administered in person to residents and distributed randomly through Google Forms at various points within the study area. Participation was voluntary, and assistance was provided to those who requested it.

Due to time and resource constraints, the survey was conducted using a mobile device with Internet access. For elderly participants, assistance was offered through reading and collaboratively answering the questions.

### 2.4. Validation and Internal Consistency

The survey was validated through expert judgment using Hernández Nieto’s validity coefficient [18]. The internal reliability was assessed with a 10% sample of the total required, using Cronbach’s alpha and McDonald’s omega coefficients.

The survey for data collection underwent a rigorous evaluation process, involving three external experts. These specialists, who were not involved in the development of the survey, contributed expert judgments and critical evaluations measuring adequacy and relevance. Consequently, the questions underwent iterative reviews and adaptations until a content validity coefficient was achieved, which was established at 0.84. This meticulously calculated indicator reflects both the adequacy and relevance of the questions with regard to the research objectives [18].

The content validity coefficient, according to Hernández Nieto, ranges from 0 to 1. A coefficient close to 0 indicates low validity, meaning that the items are less relevant to the construct being measured. A coefficient between 0.50 and 0.69 signifies moderate validity, suggesting that the items are somewhat relevant but may need refinement. Values between 0.70 and 0.89 indicate good validity, showing that items are well aligned with the construct. A coefficient of 0.90 or higher represents excellent validity, indicating a high degree of relevance and adequacy of the items. With a coefficient of 0.84, the survey questions demonstrate a high level of content validity, confirming their strong alignment with the research objectives [19].

Following the design and validation phase of the questionnaire, a pilot test with 10% of the selected sample was conducted to assess its practical performance. During this stage, an analysis of indicators of the instrument’s internal consistency was performed. These indicators are crucial to ensure the reliability and consistency of the responses collected through the survey.

Cronbach’s alpha coefficient, a widely accepted measure of internal consistency, is used to assess correlations between survey questions. In our study, the score of 0.79 indicates substantial consistency among responses, confirming the homogeneity of the questions and their ability to consistently measure the construct. Simultaneously, McDonald’s omega index, which more comprehensively addresses the multidimensionality of scales, yielded a robust 0.81, further reinforcing the reliability of the survey in terms of internal consistency [20,21].

The results obtained revealed a noteworthy score, reaching a robust result in Cronbach’s alpha coefficient and in McDonald’s omega index (Table 2).

In general, the correlations between items are positive, indicating that the questions are related to each other. However, there are some questions with weak correlations (<0.3). These were retained due to interest in obtaining the necessary information for the study, as it is necessary to assess whether the impact of land use change affects the sighting of CD vectors.

### 2.5. Data Analysis

All statistical analyses were performed using Jamovi software (The Jamovi Project 2024; jamovi Version 2.5 [computer software]; retrieved from https://www.jamovi.org, accessed on 23 September 2024). A descriptive analysis was conducted for the questions, providing an overview of the data distribution. For the inferential phase, the Chi-square statistic (χ^2^) was used to explore the relationships between variables, including vector sightings reported by the respondents. The indicator of vector sightings was determined through specific questions included in the survey, and these sightings were treated as a dichotomous and qualitative indicator.

## 3. Results

### 3.1. Knowledge Related to CD

The data collected from the applied survey correspond to a sample of 383 individuals. The average age of the respondents was 17 years, with a standard deviation of 5 years. The youngest person in the sample was 14 years old, and the oldest was 66 years old. The first quartile (Q1) age was 15 years, the median age was 16 years, and the third quartile (Q3) age was 17 years. This distribution suggests that most respondents were relatively young, with a broad range extending to older ages. Of the participants, 54.8% were women and 45.2% were men. Additionally, a significant 81.2% of respondents had lived in Izucar de Matamoros, Puebla, for 10 years or more.

Regarding knowledge of CD, only 27.4% of the respondents knew what CD is, and only 3.7% had participated in any awareness campaign, whether at work, at school, or by the government. A total of 83.3% of the inhabitants of the study locality had a pet that met the characteristics that allowed them to be considered reservoirs, such as a dog, cat, or stable animal.

In the data collection, total participation from 47 neighborhoods in the town of Izucar de Matamoros was achieved. With the information collected, it was confirmed that awareness in these localities and the population’s participation rate in awareness campaigns barely reached 3.7%. Of these 3.7%, only 0.5% were attributed to government campaigns, while the rest corresponded to campaigns in schools (1.8%) and at work (1.3%); in all, 27.4% of the inhabitants knew what CD is.

An important fact is that 47.1% of the inhabitants of Izucar de Matamoros have managed to notice the vectors, either inside their homes or in the areas surrounding them. Similarly, it was confirmed that 83.3% of the respondents have some domestic animals.

### 3.2. Importance of Housing Construction Materials and Their Correlation with Triatomine Presence

In the case of the construction materials used for the walls of the respondents’ houses, structures of lower quality (category “C”: straw, adobe, sheet metal, or wood) were observed to have a higher rate of intradomiciliary sightings of the vectors, with 57% of cases confirmed. On the other hand, walls with plastered brick (category “A”) and those without plaster (category “B”) showed lower percentages, with 9% and 33% of sightings, respectively.

Regarding roofing materials, the trend was similar. Lower-quality roofs (category “C”: straw, grass, or similar) were associated with 73% of intradomiciliary sightings of the vector, while concrete roofs (category “A”) and roofs made of tiles, wood, or sheet metal (category “B”) showed lower percentages, with 9% and 17% of sightings, respectively. This supports the hypothesis that the quality of the roofing material can influence the presence of the CD vector.

When the material characteristics of the floors were analyzed, once again, an association was observed between the quality of the material and the presence of the vector. Lower-quality floors (category “C”: wood, earthen floor, or other materials) were recorded in 37% of intradomestic sightings of the vector, while concrete and tile floors (category “A”) and concrete floors (category “B”) showed lower percentages, with 17% and 6% of sightings, respectively.

### 3.3. Environmental Conditions and the Distribution of Triatomines

Regarding changes in land use, it was observed that areas with the construction of houses, businesses, and paving (category “A”) showed a 53% vector sighting rate, while areas with tree cutting for the use of wood or the utilization of other natural resources (category “B”) presented a concerning 68% sighting rate. On the other hand, garbage dumps and land use changes for agriculture (category “C”) were associated with a sighting rate of 52%.

In the context of seasonal sightings, the results showed that during spring–summer (category “A”), there was an alarming 98% rate of vector sightings, compared to summer–fall (94%) and fall–winter (85%).

These preliminary observations, expressed as percentages, emphasize the importance of considering construction materials when addressing CD prevention. However, this analysis is descriptive and does not establish causality. Several studies have shown that building materials can encourage the presence of triatomines in a peridomiciliary and intradomiciliary manner. Furthermore, it has been observed that certain types of triatomines, such as *Meccus pallidipennis*, can adapt to good building materials such as so-called raw cement [22].

Subsequently, through the Chi-square statistic, it was demonstrated that the type of roof material plays a decisive role in intradomiciliary sightings of CD vectors (Table 3). Roofs made of materials such as straw, palm, shingles, tiles, wood, and sheet metal have a high rate of vector infestation (χ^2^ = 55.69, df = 2, *p* < 0.001, Cramér’s V = 1.06, CI = 95%).

A relationship was also demonstrated with the characteristics of the type of housing, with the most significant impact on intradomiciliary sightings attributed to the type of wall material (χ^2^ = 34.73, df = 2, *p* < 0.001, Cramér’s V = 0.83, CI = 95%), followed by the floor material (χ^2^ = 6.43, df = 2, *p* = 0.04, Cramér’s V = 0.36, CI = 95%).

In this study, the role of seasonality in vector sightings within homes or in the vicinity of residents’ houses was also questioned. This section of inferential statistics showed a weak association (χ^2^ = 2.63, df = 2, *p* = 0.27, Cramér’s V = 0.23, CI = 95%), and sightings related to changes in land use did not exhibit an association with CD vector sightings.

## 4. Discussion

The results obtained in this study provide detailed information on community participation, awareness of CD, and factors associated with triatomine sightings in Izucar de Matamoros, Puebla.

The low participation rate in awareness campaigns reflects a significant lack of knowledge about this disease, complicating its prevention. It is highly important that the government, in collaboration with the health sector, implements awareness campaigns through various channels, such as television and radio advertisements, social media, and educational programs in schools and communities. This information should be clear, accessible, and tailored to different socioeconomic groups and be distributed through various channels to reach all residents in endemic areas, as was done in Brazil [23].

Particularly concerning is that only 0.5% of the participation came from government campaigns, indicating very little interest and confirming that, in this area, CD remains an NTD of little concern to the health sector. This translated into 72.6% not being aware of the disease and its clinical importance. It should be noted that knowledge of CD serves as the first barrier to halting its transmission.

In this study, the statistical analysis revealed, based on the establishment of a positive relationship, that the type of roof material is a determining factor of intradomiciliary sightings of vectors. Roofs made of materials such as thatch, palm, shingles, tile, wood, and sheet metal show a high rate of vector infestation, suggesting the need for specific interventions in homes with these materials, as seen in previous works [24,25,26].

The significant relationship between CD vector sightings and housing characteristics, especially wall materials, and, to a lesser extent, floor materials, highlights the importance of addressing housing construction as a crucial aspect of preventing CD. Considering that 70.3% of the rural population of Izucar de Matamoros live in poverty, we can infer that a high percentage of residents are exposed to acquiring CD due to their quality of life determined by their type of housing [27].

The high percentage of residents who have observed the presence of the vectors inside their homes or in nearby areas underscores the need for more effective prevention strategies. Socioeconomic factors related to housing type are confirmed to be a determinant in intradomiciliary sighting of the CD vectors, as shown in previous studies [5,25,28], given the adaptability of these bugs to intradomiciliary life.

The high prevalence of respondents with domestic animals highlights the importance of including vector control strategies in environments with an animal presence, as demonstrated in various studies [29,30]. Domestic animals such as dogs or cats serve as key reservoirs, increasing the risk of acquiring CD in exposed populations [31,32]. In many situations, they act as a link between domestic and sylvatic reproductive cycles by not staying indoors all the time and serving as natural reservoirs for this disease; moreover, dogs can be used as important sentinels to determine the risk of *T. cruzi* infection [33].

In this study, we also investigated whether seasonality played a significant role in vector sightings, either inside households or in the vicinity of residents’ homes in the study area. Despite the weak association found in the seasonal analysis, sightings of CD vectors were observed to be more likely during the spring–summer period. However, it is important to note that a more in-depth study is needed due to the limitations presented in this work.

Despite the demonstrated mild association, as reported in Gorla’s work in 2021, CD vectors, being cold-blooded organisms, are influenced by ambient temperature in terms of their physiological and population responses, especially regarding reproduction, survival, and dispersion. If the temperature were to increase due to climate change, there is a possibility that CD vectors could expand geographically into areas that were previously too cold for their species’ needs. Similarly, an increase in the metabolic rate of parasites could shorten their infectious periods, resulting in a potential increase in cases [34].

Similarly, as reported in the literature in the work of Tamayo et al., an increase in temperature favors the development and fecundity of the *Rhodnius prolixus* vector, resulting in an increase in *T. cruzi* transmission and, consequently, the probability of CD infection [35]. This is due to the shortening of the bug’s maturation period, an increase in vector fecundity, a shorter egg-hatching time, and an increase in the infective forms present in the bug’s feces.

On the other hand, changes in land use did not show an association. A similar result was obtained by Penados et al.; in their study, it was discovered that changes in land use due to anthropogenic activity for the exploitation of natural resources did not impact CD vector sightings [36]. However, Gottdenker et al., in their study on the identification of *Rhodnius pallescens* abundance, confirmed this association [37]. Despite changes in land use due to anthropogenic activity for the exploitation of natural resources, there was no impact on sightings of the CD vectors. Socioeconomic factors, such as housing quality, remain the determinants of vector detection within households in CD endemic areas.

In this study, we evaluated whether the impact of the change in land use due to human activities favored the sighting of CD vectors in the surroundings of the residents of Izucar de Matamoros, Puebla. Anecdotally, the impact of changes in land use was not statistically significant and could not support the hypothesis in question.

CD remains a significant and complex public health problem in Mexico. The prevention of CD requires a multifaceted approach involving the government, the community, and public health sectors with a global scope, considering the population’s needs, to positively impact the outlook regarding CD in endemic areas such as Izucar de Matamoros, Puebla.

### Limitations

This study had limitations including incomplete data for variables like seasonality, resulting in information loss. The relationship between triatomine sightings and seasonality was based on the memory of the people interviewed. The period in which the surveys were conducted was during the winter and spring seasons. The survey should also be conducted in summer and autumn to ensure sightings dependent on the season. Also, this study relied on sightings of triatomines that were identified by showing images to the people surveyed. To ensure the presence of triatomines in houses, collections of triatomines should be carried out, although this is sometimes not possible due to a lack of permission from people to enter their houses. Nevertheless, this study advanced our understanding of the relationship between poor-quality housing materials and triatomine sightings.

In general, efforts were made to avoid non-sampling errors to prevent data bias. However, it should be noted that most of the interested respondents were young, as adults and the elderly were less inclined to participate.

Due to certain limitations, it was not possible to implement a block sampling method across the entire study area. These limitations included resource constraints, time restrictions, and logistical challenges, which affected the ability to comprehensively apply this sampling technique. Consequently, this study focused on alternative methods to ensure representative data collection, but the absence of full block sampling may have influenced the overall scope of the findings.

## 5. Conclusions

The economic factors related to housing quality allowed us to identify that houses built with lower-quality materials are associated with a higher number of intradomiciliary sightings. The combination of lower-quality walls (category “C”: straw, adobe, sheet metal, or wood), lower-quality floors (category “C”: wood, earthen floor, or other materials), and lower-quality roofs (category “C”: straw, grass, or similar) was associated with the highest index of sightings [38,39]. Seasonality showed a weak association, and a lack of a relationship with land use change was found.

In conclusion, these findings emphasize the need for more effective awareness strategies, more active government involvement, and specific measures to address housing conditions that favor vector infestation. Interdisciplinary collaboration and ongoing adaptation of strategies are crucial for achieving effective control of CD in the Izucar de Matamoros region; it is a complex problem that, in turn, requires complex solutions. Additionally, we consider that this problem is not exclusive to this region of Puebla. In Mexico, there are buildings with low-quality materials, and in Latin America, there are also houses with low-quality construction materials, so this would be a recurring problem in several countries and must be addressed to avoid interactions with triatomines.

## Figures and Tables

**Table 1:** Variables using the chi-square statistic.

Variable	Categories
roof	A: concrete roof
	C: thatch, grass, or similar roof
	B: tile, wood, or sheet metal roof
floor	A: concrete and tile floor
	B: concrete floor
	C: wood, dirt, or other material floor
wall	A: plastered brick wall
	B: unplastered brick wall
	C: thatch, adobe, sheet metal, or wood wall
seasonal sighting	A: spring–summer
	B: summer–fall
	C: fall–winter
land-use change	A: construction of houses, businesses, paving, etc.
	B: tree felling for wood use or utilization of other natural resources
	C: garbage dumps, land use change for agriculture, etc.

**Table 2:** Reliability analysis of survey questions: correlations, Cronbach’s α, and McDonald’s ω.

		Correlation if the Question is Discarded	
	Correlation of the Question with Others	Cronbach’s α	Mc’Donald’s ω
1. Wall of house: What is the predominant material?	0.5582	0.776	0.795
2. Roof of house: What is the predominant material?	0.3630	0.788	0.806
3. House on the ground floor: What is the predominant material?	0.5265	0.775	0.794
4. How long have you been living in Izucar de Matamoros? ᵃ	0.1336	0.801	0.817
5. Have you seen any of these insects in your area? (shown photo 1)	0.3456	0.788	0.803
6. Have you seen any of these insects in your area? (shown photo 2)	0.6375	0.773	0.786
7. Have you seen any of these insects in your area? (shown photo 3)	0.6601	0.773	0.783
8. Sightings in the yard	0.5298	0.776	0.794
9. Sightings in the garden	0.4369	0.782	0.802
10. Inter-domiciliary sightings	0.4405	0.783	0.799
11. How often have you seen it?	0.6491	0.762	0.780
12. Do you have pets?	0.1705	0.796	0.814
13. Have you noticed when you saw these insects?	0.4966	0.787	0.796
14. Change in land use for urbanization ᵃ	0.0150	0.803	0.821
15. Change in land use for natural resource utilization	0.1037	0.797	0.814
16. Change in land use for urban waste disposal	0.1037	0.797	0.814
17. Have you ever participated in any CD campaign? ᵃ	0.4483	0.786	0.800
18. Through which broadcast media? ᵃ	0.3656	0.787	0.803
19. Would you like to participate in a CD broadcasting diffusion?	0.1061	0.0805	0.818

ᵃ Reverse-scored question. “Correlation if the Question is Discarded” shows how the internal consistency of the survey would change if the questions were removed, with higher correlations indicating better consistency. “Correlation of the Question with Others” measures how well the question aligns with the rest of the survey. Cronbach’s α assesses internal consistency, with values near 1.0 indicating high reliability. McDonald’s ω, often more robust than Cronbach’s α, also shows high reliability in this case. Both indicators demonstrate good consistency in the survey.

**Table 3:** Summary of Chi-square and Cramér’s V statistics for vector sightings and associated factors.

Variable	χ^2^	*p*-Value	Cramér’s V
Roof Material	55.69	<0.001	1.06
Wall Material	34.73	<0.001	0.83
Floor Material	26.47	<0.001	0.73
Seasonal Sighting	6.43	0.04	0.36
Land Use Change	2.63	0.27	0.23

χ^2^—Chi-square. The critical value result is x^2^(1 − α)(r − 1)(c − 1) = 5.99: calculated critical value; Ho: independence of variables; Hi: related variables. If x^2^ > x^2^(1 − α)(r − 1)(c − 1), then Hi is accepted, and it indicates that an association was found. Note: The degrees of freedom for all Chi-square tests in this table are df = 2. For Cramér’s V: Results greater than or equal to 0.3 indicate a moderate association, while values equal to or greater than 0.6 indicate a strong association between variables.

## Data Availability

The data used to support the findings of this study are included within the article. If anyone is interested in the raw data (Excel), they are available from the corresponding authors upon request.

## Supplementary Materials

The following supporting information can be downloaded at: https://www.mdpi.com/article/10.3390/diseases12100238/s1, Figure S1. Informed consent request and survey that were distributed among residents of a community near Puebla with a prevalence of triatomines.
