# Supplementary material for: The Impact of Environmental and Housing Factors on the Distribution of Triatominae (Hemiptera, Reduviidae) in an Endemic Area of Chagas Disease in Puebla, Mexico
Source: Diseases. 2024 Oct 2;12(10):238. doi: 10.3390/diseases12100238 (PMC11506842; doi:10.3390/diseases12100238)
Supplement: Supplementary file 1 [file diseases-12-00238-s001.zip › diseases-3161357-supplementary.pdf]

"Evaluación del Impacto ambiental en la diseminación y proliferación de insectos Triatominos (chinches besuconas) en la región de Izúcar de Matamoros, Puebla, México"

Encuesta para recabar información en un estudio por parte de la BUAP

* Indica que la pregunta es obligatoria

1. CONSENTIMIENTO INFORMADO DEL PARTICIPANTE Nombre del estudio: "Evaluación del Impacto ambiental en la diseminación y proliferación de insectos Triatominos (chinches besuconas) en la región de Izúcar de matamoros, Puebla, México". Somos un equipo de investigadores formado por profesores y estudiantes del Posgrado en Ciencias Ambientales y profesores del Instituto de Ciencias, ambos pertenecientes a la Benemérita Universidad Autónoma de Puebla, los integrantes pertenecen a grupos de investigación oficialmente reconocidos en materias como la Prevención de Riesgos por insectos, Hábitos Saludables y Métodos y Técnicas de Investigación en Ciencias de la Salud. Estamos realizando un estudio en México, para conocer el impacto ambiental y como repercute en la movilización de insectos potencialmente peligrosos para la comunidad, debido a que pueden ser portadores de Trypanosoma cruzi, un parásito sanguíneo que puede generar diversas complicaciones en la salud de las personas. La información obtenida, nos permitirá identificar factores ambientales fundamentales en la movilización de estos insectos para proponer medidas de prevención adecuadas. Para ello, necesitamos que los/as participantes contesten a este cuestionario del que extraeremos la información. Usted debe saber que: 1) Su participación en este estudio es totalmente voluntaria. 2) La participación o no participación no supondrá para usted ningún beneficio ni perjuicio. 3) Todos los datos obtenidos en este estudio serán confidenciales 4) La información obtenida, únicamente se utilizará para los fines específicos del estudio. 5) Una vez que comience a contestar el cuestionario, es libre de abandonar el estudio en cualquier momento, no suponiendo ningún tipo de consecuencias para usted. Si lo desea, puede contactar con las siguientes personas, como referentes del proyecto: Dra. Alia Mendez Albores. Responsable del proyecto, Posgrado en Ciencias Ambientales. Benemérita Universidad Autónoma de Puebla (México). e-mail: aliamendez@correo.buap.mx Dr. Alejandro Carabarín Lima. Responsable del proyecto, Instituto de Ciencias, Benemérita Universidad Autónoma de Puebla (México). E-mail: alejandro.carabarin@correo.buap.mx Miguel Ortega Caballero. Responsable del proyecto, Instituto de Ciencias, Benemérita Universidad Autónoma de Puebla (México). E-mail: miguel.ortegacaballero@viep.com.mx Usted declara que (debe marcar las dos opciones): *

Selecciona todas las opciones que correspondan.

- [ ] Es mayor de 15 años
- [ ] Acepta participar en este estudio

2. 1.- ¿Vives en Izúcar de Matamoros? *

Marca solo un óvalo.

- ◯ si
- ◯ no

3. 2.- ¿En qué colonia te encuentras viviendo? *

Marca solo un óvalo.

- Amatitlanes
- Campestre la Paz
- Campo Nuevo
- Colucan
- Cruz Verde
- Cruz Verde 2a Sección
- Del Empleado
- El Calvario 1a. Sección
- El Calvario 2a Secc
- El Jardín
- El Mangal
- Guadalupe
- Itzocan
- Izúcar de Matamoros Centro
- La Asunción
- La Curva
- La Magdalena
- La Paz
- Las Espigas
- Las Palmas
- Las Violetas
- Lázaro Cárdenas
- Lomas de Alchichica
- Los Ahuehuetes
- Los Arcos
- Los Fresnos
- Los Fresnos FOVISSSTE
- Los Reyes
- Mazatla
- Morelos
- Niños Héroes
- Paseos de Matamoros
- Rancho Juanitos
- Residencial el Mangal
- Residencial la Asunción
- San Bernardino
- San Diego Chiconcuac
- San Juan Coahuixtla

- San Juan Piaxtla
- San Martín Huaquechula
- San Miguel Izucar
- Santa Catarina
- Santa Cruz Coatla
- Santa Cruz Tecoxco
- Santiago Mihuacan
- Santo Tomás
- Valle Primavera
- Villa Pao
- Villas Matamoros
- Villas Reales
- Vista Hermosa
- Ayutla
- San nicolás
- Matzaco
- San Carlos
- Raboso
- Otro

4. 3.- Sexo

Marca solo un óvalo.

- Masculino
- Femenino

5. 4.- Edad: *

Marca solo un óvalo.

- 13
- 14
- 15
- 16
- 17
- 18
- 19
- 20
- 21
- 22
- 23
- 24
- 25
- 26
- 27
- 28
- 29
- 30
- 31
- 32
- 33
- 34
- 35
- 36
- 37
- 38
- 39
- 40
- 41
- 42
- 43
- 44
- 45
- 46
- 47
- 48
- 49
- 50

- 51
- 52
- 53
- 54
- 55
- 56
- 57
- 58
- 59
- 60
- 61
- 62
- 63
- 64
- 65
- 66
- 67
- 68
- 69
- 70
- 71
- 72
- 73
- 74
- 75
- 76
- 77
- 78
- 79
- 80
- 81
- 82
- 83
- 84
- 85
- 86
- 87
- 88
- 89
- 90
- 91

- 92
- 93
- 94
- 95
- 96
- 97
- 98
- 99
- 100 o mas

6. 5.1.- Paredes ¿Cúal es el material predominante en la casa? *

Marca solo un óvalo.

- Pared de ladrillo revocado
- Pared de ladrillo sin revoque
- Pared de paja, adobe, lamina o madera

7. 5.2.- Suelo ¿Cúal es el material predominante en la casa? *

Marca solo un óvalo.

- Suelo de concreto
- Suelo de concreto y azulejo
- Suelo de madera, Suelo de tierra u otro material

8. 5.3.-Techo ¿Cúal es el material predominante en la casa? *

Marca solo un óvalo.

- Techo de concreto
- Techo de tejas/madera o lamina
- Techo de paja, zacate, suyate, manaca u hoja de caña

9. 6.- ¿Cuánto tiempo tienes viviendo en Izúcar de Matamoros, Puebla? *

Marca solo un óvalo.

- menos de 1 año
- 1 año
- 2 años
- 3 años
- 4 años
- 5 años
- 6 años
- 7 años
- 8 años
- 9 años o mas

10. 7.- ¿Sabes qué es la enfermedad de Chagas? *

Marca solo un óvalo.

- Si
- No

11. 8.- ¿Conoces a alguien que viva con la enfermedad de Chagas? *

Marca solo un óvalo.

- Si
- No

12. 9.- De la siguiente lista, ¿Cuáles son los síntomas de la infección primaria de la enfermedad de chagas? *

Marca solo un óvalo.

- Los síntomas pueden oscilar entre leves y graves, aunque muchas personas no presentan síntomas hasta que llegan a la etapa crónica.
- Los síntomas incluyen rigidez o dolor en el pecho, el cuello, la espalda o los brazos, así como fatiga, mareos, ritmo cardíaco anormal y ansiedad.
- Por lo general, el primer síntoma es picor o dolor de garganta, o molestias en la nariz.
- Ninguna de las anteriores.

13. 10.- ¿Conoces las vías de transmisión de dicha enfermedad? *

Marca solo un óvalo.

- si
- no

14. 11.- ¿Has visto alguno de estos insectos en tu localidad? *

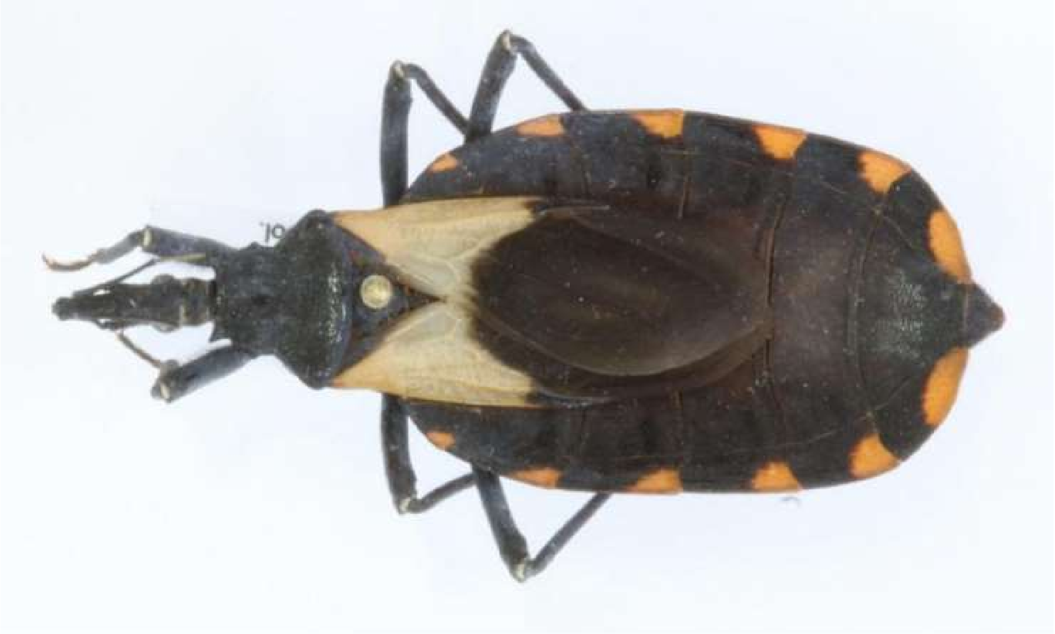

Marca solo un óvalo.

- si
- no

15. 12.- ¿Has visto alguno de estos insectos en tu localidad? *

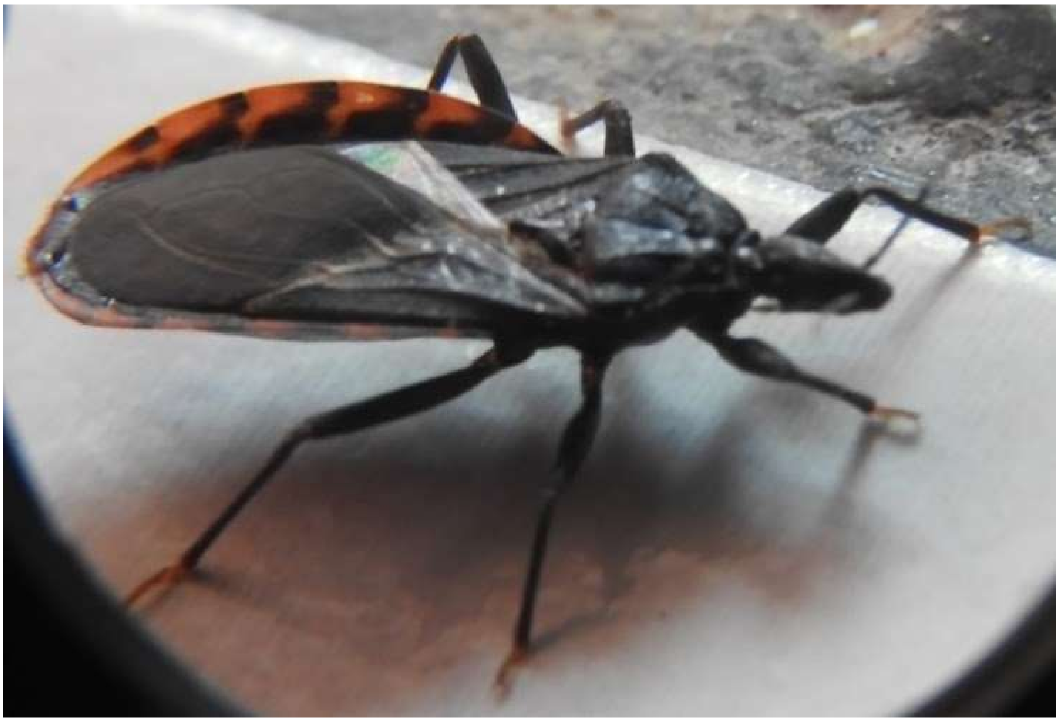

Marca solo un óvalo.

- ⬭ si
- ⬭ no

16. 13.- ¿Has visto alguno de estos insectos en tu localidad? *

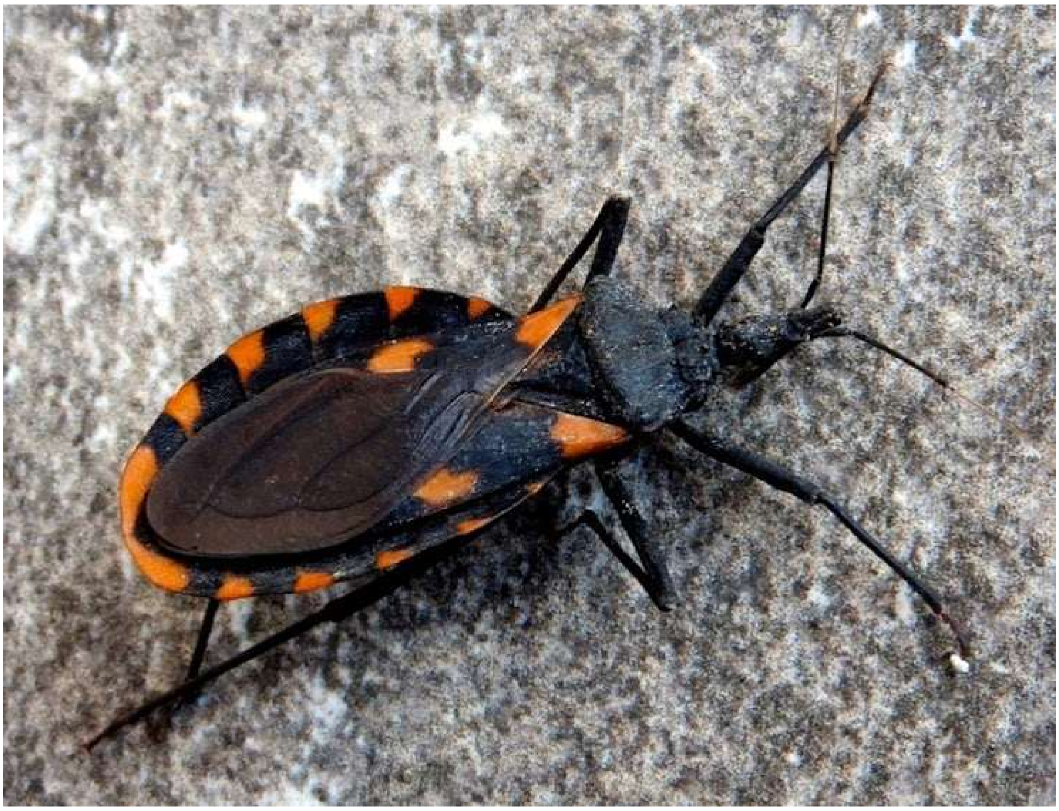

Marca solo un óvalo.

- si
- no

17. 14.- Si llegaste a identificar alguno de los insectos señala en dónde [Selecciona tantas como sea necesario] *

Selecciona todas las opciones que correspondan.

- [ ] En el interior de mi domicilio
- [ ] En el patio/jardín
- [ ] En la calle
- [ ] No lo he visto

18. 15.- ¿Con qué frecuencia lo has visto? *

Marca solo un óvalo.

- Menos de una vez al año
- Más de una vez al año
- No lo he visto

19. 16.- ¿Has notado en que momentos has visto a estos insectos con mayor frecuencia? *

Marca solo un óvalo.

- Primavera-verano
- Verano-otoño
- Otoño-invierno
- No lo he visto

20. 17.- ¿Tienes alguna mascota o animal de granja? [perro,gato caballos,vacas,burros, conejos] *

Marca solo un óvalo.

- si
- no

21. 18.- ¿Cerca de tu domicilio se han presentado obras urbanas? [construcción de nuevos asentamientos, tiraderos de basura, casa, etc.] *

Marca solo un óvalo.

- si
- no

22. 19.- Menciona que tipo de asentamientos: *

Marca solo un óvalo.

- Obras Urbanas: construcción de casas, negocios, pavimentación, etc.
- Tiraderos de basura, cambio de uso de suelo para la agricultura, etc.
- Tala de árboles para utlización de madera o aprovechamiento de otros recursos naturales

23. 20.- ¿Has participado en alguna campaña de concientización de dicha enfermedad? *

Marca solo un óvalo.

- Si
- no

24. 21.- Si tu respuesta anterior fue "si": ¿Dónde has participado en dicha campaña? *

Marca solo un óvalo.

- Escuela
- Trabajo
- Campañas de gobierno/ayuntamiento
- Otra
- No he participado

25. 22.- ¿Te gustaría participar en alguna campaña de concientización de dicha enfermedad?

Marca solo un óvalo.

- Si
- No

26. 23.- Si tu respuesta fue si: ¿Por qué medio te gustaría participar?

Marca solo un óvalo.

- Plática
- Tripico informativo
- Correo electrónico
- Cartel informativo

Google no creó ni aprobó este contenido.

Google Formularios
